# Tactile Sensing During Backward Locomotion in the Mole Cricket

**DOI:** 10.3390/insects17060564

**Published:** 2026-05-29

**Authors:** Avi Amir, Omer Yuval, Kobi Fuxman, Dafna Cohen, Amir Ayali

**Affiliations:** 1 School of Zoology, Tel Aviv University, Tel Aviv 6997801, Israel; aviamir@mail.tau.ac.il (A.A.); omeryu@tauex.tau.ac.il (O.Y.); kobifuxman@mail.tau.ac.il (K.F.); dafnacohen1@mail.tau.ac.il (D.C.); 2 Sagol School of Neuroscience, Tel Aviv University, Tel Aviv 6997801, Israel

**Keywords:** mole cricket, backward walking, mechanosensation, subterranean locomotion, tactile sampling

## Abstract

Subterranean locomotion challenges animals to maintain orientation and efficiently navigate confined spaces where vision is limited and local geometry is uncertain. Mole crickets (Gryllotalpidae), which regularly travel through self-excavated tunnels, provide a useful model for studying mechanosensory control of locomotion under these conditions. We hypothesized that backward walking, a prominent component of the mole cricket’s behavioral repertoire, is supported by complementary tactile input from the antennae and cerci. Adult *Gryllotalpa tali* were filmed while walking forward and backward in a narrow, straight tunnel arena under red light using high-speed video. Markerless pose tracking was used to quantify antennal and cercal tip movements, orientations, and wall-contact events in body and arena coordinates. Backward walking produced clear changes in tactile sampling behavior: antennae were reoriented and extended more posteriorly, while lateral cercal movements increased sensory coverage and posterior tactile input. Wall-contact monitoring suggested more frequent touching during backward locomotion by the antennae. These findings indicate that mole crickets adaptively reorganize active and passive mechanosensory sampling when moving backward, potentially improving boundary detection, stabilizing body posture, negotiating tunnel constraints, and supporting locomotion-related decision making during locomotion in tunnels.

## Simple Summary

Moving underground is difficult because animals cannot rely much on vision and must constantly sense the environment around them. Mole crickets are a good example because they often move through the tunnels that they dig themselves. This study looked at how mole crickets manage to walk backward in such tight spaces. Researchers recorded adult mole crickets walking forward and backward in a narrow tunnel and tracked the movement of two pairs of sensory body parts: the antennae at the front and the cerci at the rear. When the mole crickets moved backward, their antennae shifted to reach farther back, while the cerci moved more from side to side, increasing the area they could sense. The insects also touch the tunnel walls more often when walking backward. These results suggest that mole crickets adjust how they gather touch information depending on the direction they move. This flexible sensing system helps them stay oriented, avoid collisions, and move safely through narrow underground tunnels.

## 1. Introduction

**Figure 1:**
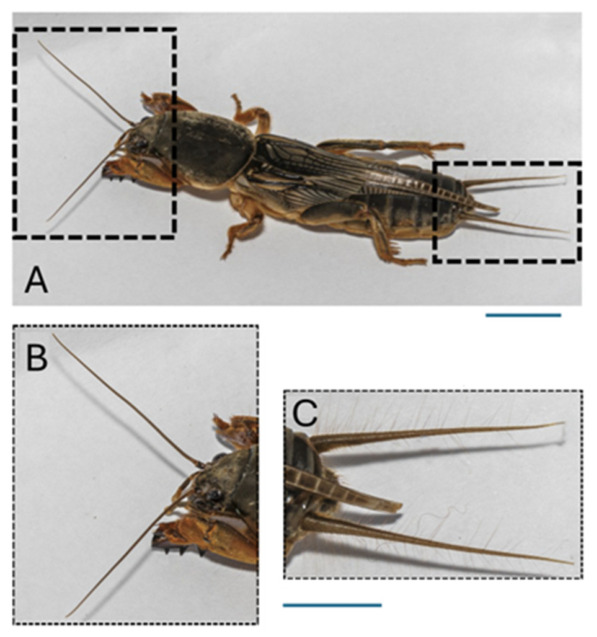
The mole cricket (**A**) and its sensory appendages: antennae (**B**) and cerci (**C**). Scale bar in (**A**)—1 cm; in (**B**,**C**)—0.5 cm.

Subterranean environments present distinctive sensorimotor challenges: animals must move and orient in confined, cluttered spaces where vision is unreliable or absent, and knowledge of the local geometry is limited. These conditions may drive specialized adaptations in sensory–neural control of movement, with increased investment in non-visual modalities and near-field exploratory appendages [1,2]. Mole crickets (Gryllotalpidae; Figure 1) are a compelling model for subterranean locomotor control because their lifestyle is tightly coupled to burrow construction and movement within self-excavated tunnels [3,4,5]. Hence, in these insects, mechanosensation is expected to be tightly linked to locomotor control.

Insect nervous systems integrate mechanosensory feedback with motor commands to maintain stability, negotiate uneven environments, and flexibly alter movement patterns [6,7]. Tactile sensing is commonly mediated by mechanoreceptive sensilla distributed across the body and appendages [8] and can be deployed passively through contact imposed by the environment, or actively through body or sensor motion deliberately generated to acquire information [9].

In mole crickets (Figure 1), as in other insects, the antennae are a principal substrate for active tactile sensing: they are positioned to sample near-field geometry during walking, and they are mobile and densely covered with sensory hairs [10]. In cockroaches, antennal movements have long served as a model for active touch, illustrating how animals shape sensory input by moving the sensor to localize objects and maintain oriented locomotion relative to boundaries [11,12]. Other walking insects similarly show that antennal touch is not merely a collision detector but can be structured into exploratory “sampling” movements that drive steering decisions (e.g., ref. [13]). For example, tethered stick insects exhibit active tactile exploration and touch-triggered turning responses that link antennal contact patterns to rapid locomotor adjustments [14]. In crickets, antennal mechanosensory input supports spatial perception and modulates behavioral output, consistent with a broader role for antennae in constructing a near-field spatial representation relevant to navigation [15].

The cercal system comprises a pair of antenna-like appendages covered with mechanosensory hairs at the rear of the abdomen (Figure 1C). Cerci are classically associated with mechanosensation relevant to threats and escape [16,17] and have been studied extensively in cockroaches and crickets as a model system for sensory mapping and sensory–motor integration (e.g., refs. [18,19]). Cricket cerci are also sensitive to sound and substrate-transmitted vibrations [20,21]. A comparative phylogenetic study of tropical crickets (Orthoptera: Grylloidea) suggests that complex, diverse, and variable cerci evolved from small, hair-covered ancestral structures. Cerci were used for both air- and touch-mediated mechanoreception [22]. Cercal musculature has been described in several insects [23]. In Orthoptera, the cerci are moved by a distinct set of muscles—three abductors, a depressor, and an elevator—making the cerci maneuverable and potentially usable for tactile exploration [24]. Accordingly, cercal mechanosensation likely serves multiple behaviors depending on context, not only escape [17].

A special reference to the use of the cerci appears in Kidd’s classic study On the Anatomy of the Mole-Cricket [25]: “It is endued with the power of moving as easily in a retrograde as in a progressive direction; and, apparently to perform the office of antenna, which warn the insect of approaching danger in its progressive motions, it has two appendages, which might not improperly be called caudal antennae, evidently calculated to serve a similar purpose during its retrograde motions.” This observation also highlights the unique challenge of moving backward underground and portrays the mole cricket as a compelling model for retrograde motion.

Backward locomotion is an important component of insects’ movement repertoire [26], often recruited when forward progression is impeded, when turning is constrained, or when rapid retreat is advantageous. We have recently characterized the locomotor gaits of the mole cricket, identifying two distinct backward gaits [27,28], and further establishing this fossorial insect, which lives in networks of underground tunnels, as an intriguing model for studying the sensory control of backward locomotion.

Despite extensive knowledge about antennal touch and cercal mechanosensation in diverse insects, we still lack an integrated understanding of how these anterior and posterior mechanosensory appendages support backward locomotion, particularly in a fossorial, tunnel-dwelling specialist like the mole cricket. Here, we test the working hypothesis that backward locomotion in mole crickets is supported by complementary sensing roles of the antennae and cerci, which provide information about nearby walls and detect rearward, leading-edge contact cues. These cues may help stabilize body orientation, regulate collision avoidance, support corridor centering, and trigger direction-switch in narrow tunnels.

## 2. Materials and Methods

### 2.1. Experimental Animals

European mole crickets (*Gryllotalpa tali*) were collected in rural areas around Tel Aviv, Israel, and kept individually in glass jars with soil under dark conditions at 20–25 °C. Animals were fed flour beetle larvae, grass roots, and carrot slices. Only adults (of both sexes) were used. Individuals were video-filmed for limited durations to minimize stress. If required, brief mild tactile stimulation was used to elicit walking. Video clips were further analyzed for the temporal and spatial parameters of the sensory appendages.

### 2.2. Experimental Setup

Preliminary behavioral observations were conducted in a glass-covered 1.5 cm deep tray filled with garden soil under red light (invisible to the insects; ref. [29]). Insect locomotion was recorded in a straight and narrow tunnel-like arena (30 cm length, 2.5 cm width) with acclimation chambers at both ends. Forward and backward locomotion bouts were recorded for each animal within the same experimental session. Video was captured from above using a high-speed camera (MIKROTRON motion-Blitz cube4MGE-CM4, Unterschleißheim, Germany; lens: Vital Vision Technology Pte Ltd., Singapore), at 500 fps, at a resolution of 1280 × 1064 pixels, under red-filtered illumination. The arena geometry enabled reliable identification of wall contacts during forward and backward walking.

### 2.3. Dataset and Kinematic Analysis

Twenty insects were video-recorded in our walking setup. Among these, 10 were selected based on executing a minimum number of continuous stepping cycles, i.e., 6–14 cycles per walking bout; the mean numbers of cycles were 8.7 and 8.3 for forward and backward walking, respectively. Hence, a total of 20 high-speed video clips were analyzed, comprising one forward-walking and one backward-walking trial for each of the 10 insects. Video clips were 1 to 3 s long. Appendage kinematics were analyzed from the video using markerless pose tracking: key points were tracked on the bases and tips of both antennae and cerci, as well as anatomical landmarks defining the main body axis (the vector connecting the thorax–abdomen junction to the thorax–head junction). Data annotation, model training, and point detection were carried out using DeepLabCut 3.0 (DLC, [30]; see also [27,28]).

Appendage instantaneous orientations and angular measures were analyzed in both body-fixed and arena-fixed coordinate frames. In the body frame, sensor coordinates were translated to the thorax–abdomen junction and rotated such that the main body axis aligned with the *x*-axis. In the arena frame, sensor coordinates were translated along the long arena axis such that all thorax–abdomen junctions were aligned on this axis. To compute the bounding polygon, the “boundary” function in MATLAB 26.1 (MathWorks, Natick, MA, USA) was used. This function computes a polygon that encloses a set of predefined points. A shrinking factor of 0.01 was used; the shrinking factor controls how tightly the boundary wraps around the points, with a value of 0 yielding the convex hull.

For depicting the dynamics of sensory appendage movement during forward vs. backward walking, the angle of the vector connecting the base to the tip of the sensory appendage was calculated (in arena absolute coordinates). The circular sectors covered by each appendage in forward and backward locomotion, including the circular mean and circular standard deviation of the angles across all frames per clip, across all animals, were also analyzed. These values were calculated both in arena coordinates and relative to the animal’s body axes. Antennal angles were measured relative to the anterior/main body axis, whereas cercal angles were measured relative to the posterior body axis (such that 180° denotes straight backward).

Wall-contact events were identified by comparing appendage tip positions to the tunnel boundary. For each appendage, a wall-contact ratio was computed as the fraction of time spent in contact with the tunnel wall relative to the total duration of the locomotion bout.

### 2.4. Statistics

Paired statistical analyses (Wilcoxon signed-rank test for non-circular data, and the circular median test for circular data) were performed using within-individual comparisons between forward and backward walking. For each metric, values were averaged per individual over time within a single clip (walking bout) and then averaged across animals for each gait. Our entire data set, including all statistical analyses, can be found in the Supplementary Material.

## 3. Results

**Figure 2:**
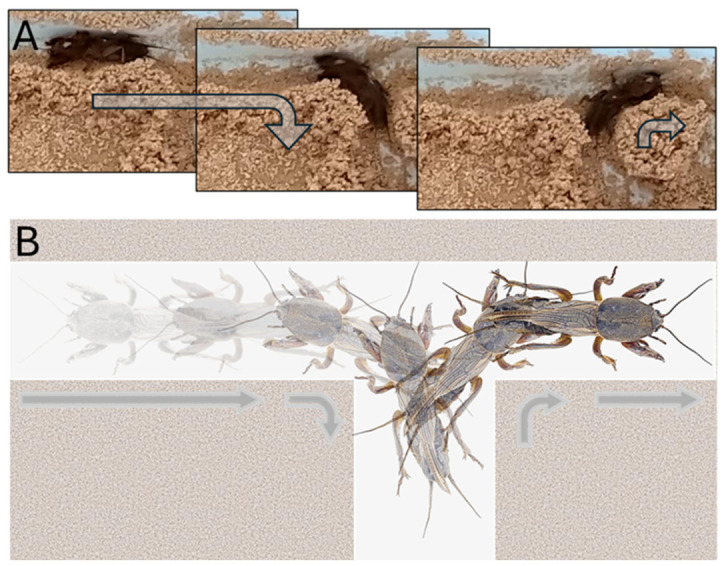
Backward locomotion in the mole cricket. (**A**) Three snapshots from a video sequence showing typical behavior in which the insect walks backward until reaching a bifurcation, which it uses to change walking direction (see arrows). (**B**) Schematic representation of the behavior shown in (**A**).

Preliminary behavioral observations of freely roaming insects revealed many instances of retrograde motion in mole crickets. The behavior depicted in Figure 2 and Video S1 was observed repeatedly, suggesting that mole crickets may detect or use sensory information about nearby environmental features during backward locomotion.

Following our hypothesis that both the antennae and the cercal system provide the insect with the tactile information needed during backward locomotion, we set out to comparatively investigate the spatial trajectories of the tips of these sensory appendages during forward vs. backward walking in our controlled experimental arena. Our entire data set, including all statistical analyses, can be found in the Supplementary Material.

**Figure 3:**
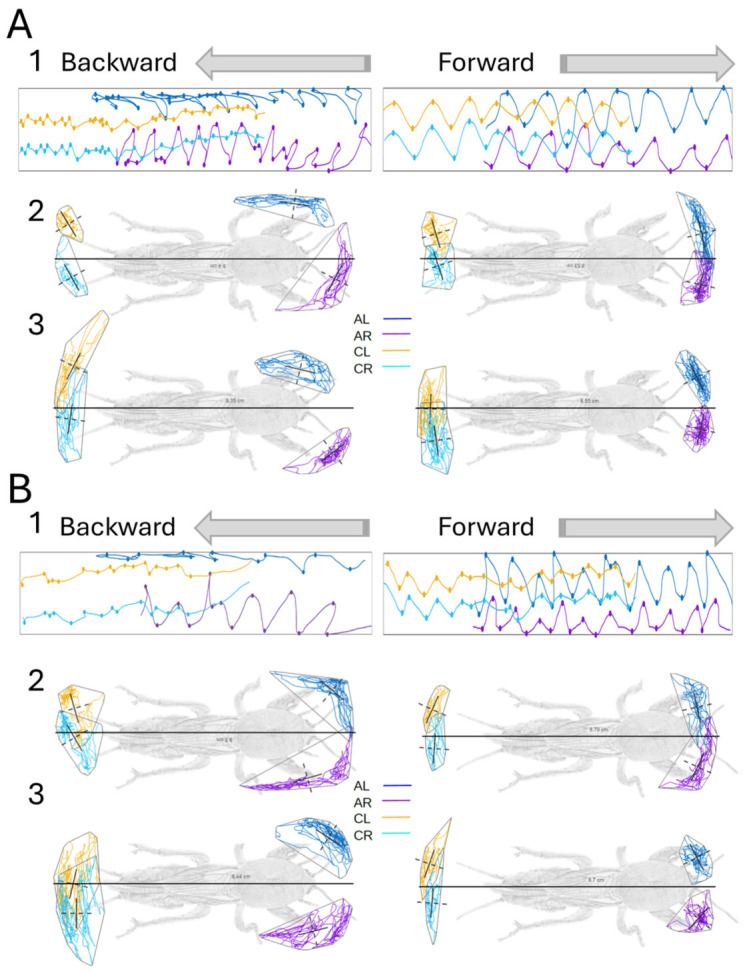
Spatial trajectories of sensory appendages during forward vs. backward locomotion. Two representative examples from two insects are shown in (**A**,**B**). In both, panel (1) depicts the dynamics of the tip positions of the sensory appendages along a walking bout, over the length of the arena. The walking direction is indicated above. In (2,3), movement cycles of the appendages are overlaid as if the insect body were tethered and did not move along the arena’s long axis. Tip positions are shown in absolute arena coordinates (2), as well as relative to the anterior body axis (3). The virtual bounding polygon made by the tips is drawn and its two major axes are noted for reference: first and second PCA component shown as solid and dashed lines, respectively. AL, AR, CL and CR denote the left and right antennae and cerci, respectively.

**Figure 4:**
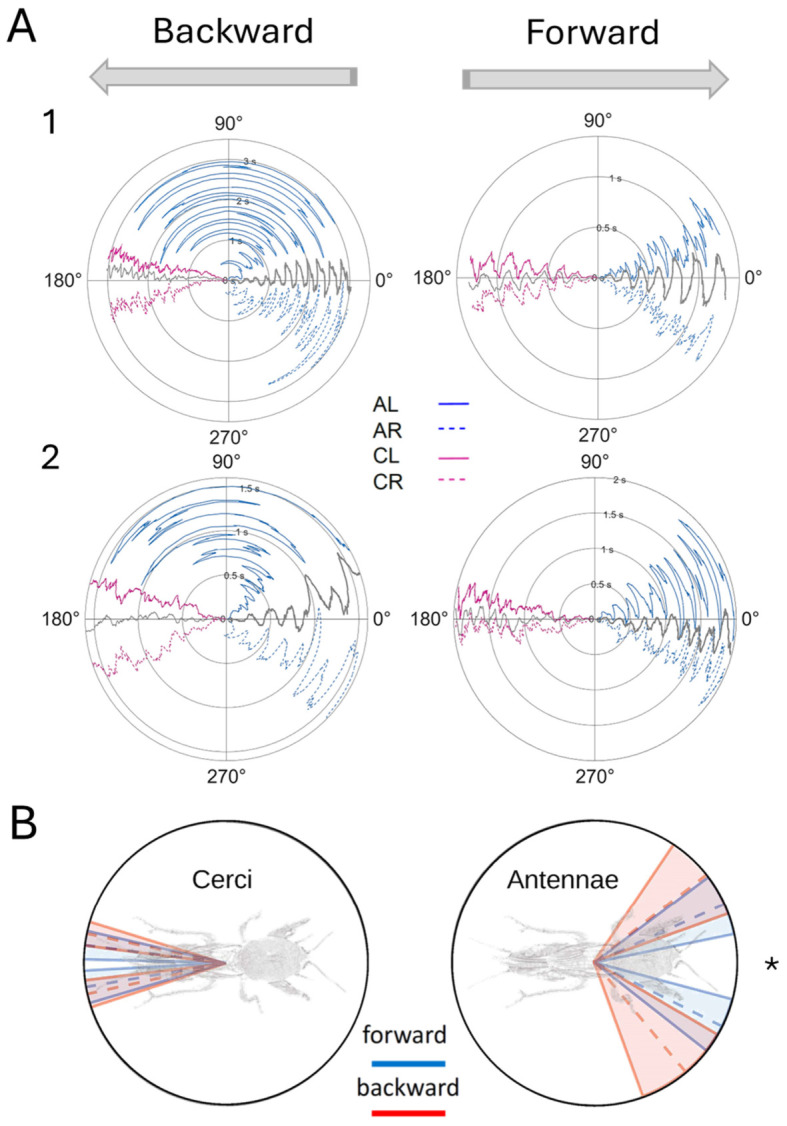
The dynamics of sensory appendage movement during forward vs. backward walking. (**A**) Two examples of forward vs. backward walking from the same individuals shown in Figure 3. The graphs depict changes during a walking bout (time shown in the radial direction) in the angle of the line connecting the base of a sensory appendage to its tip (in arena coordinates). AL, AR, CL and CR denote the left and right antennae and cerci, respectively. The changes in the angle dynamics of the anterior thorax–head and the posterior abdominal body axes are also depicted (grey lines). (**B**) Sensory appendages angular data as in (**A**), calculated relative to the major body axes. Antennal angles were measured relative to the anterior/main body axis, whereas cercal angles were measured relative to the posterior body axis. Data were averaged over all 10 insects tested for forward vs. backward walking bouts. Shaded sectors depict the average ± standard deviation. Statistical significance is indicated (* *p* < 0.05).

**Figure 5:**
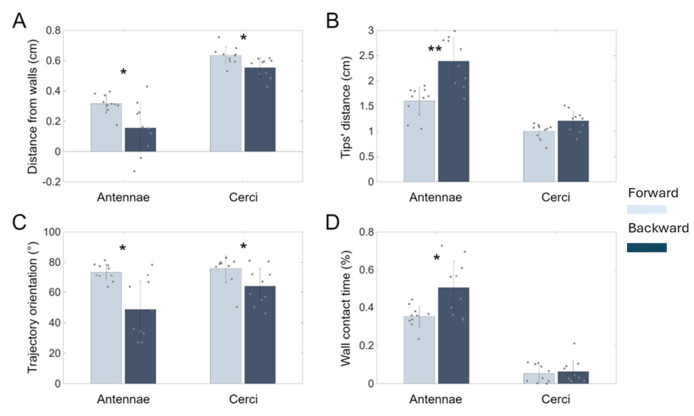
Sensory appendages in backward vs. forward locomotion. (**A**) Average distance of antenna and cerci tips from the side walls in Backward vs. forward walking (see also Figure 3A1,B1). (**B**) Average distance between the tips of the left and right sensory appendages during backward and forward walking (see also Figure 3A1,B1). (**C**) The orientation of the antenna and cerci tip overlaid trajectories in arena coordinates during Backward vs. forward walking (see Section 2. Methods; see also Figure 3A2,B2). (**D**) The percentage of time (i.e., the ratio of video frames) that the tip of the appendage touched a wall. In (**A**–**D**) dark and light shaded bars depict backward and forward locomotion, respectively. Statistical significance is indicated (* *p* < 0.05, ** *p* < 0.01).

The two examples depicted in Figure 3 and Figure 4A present several marked walking direction-dependent differences: First, throughout each walking bout, along the length of the arena, the antennal tips were overall closer to the walls during backward walking (Figure 3A1,B1; averaged across all insects in Figure 5A). Although to a lesser extent, this was also true for the cerci (see Figure 5A). Second, consistent with this pattern, the distance between the tips of the left and right antennae was larger during backward walking (see also Figure 5B). As clearly demonstrated in Figure 3, a similar trend was seen for the cerci (see also Figure 4).

The area covered by the tips of the antennae and cerci is depicted by the bounding polygon drawn around their overlaid trajectories, as shown in Figure 3 (in both arena and insect coordinates; see Section 2. Methods). This area often appeared larger in backward compared with forward walking (Figure 3 and Figure 4). The difference, however, did not reach statistical significance.

Third, a consistent difference was observed for both sensory appendages in the orientation of their overlaid tip trajectories (Figure 5C). Specifically, the antennal trajectory was strongly reoriented posteriorly (Figure 3). This difference was clear when plotting the absolute tip position (Figure 3A2,B2 and Figure 4), as well as the tip position relative to the anterior body axis (Figure 3A3,B3). In the case of the cerci, the latter captures both the passive movement (wiggling of the abdomen relative to the thorax) and active sensing motion arising from independent cercal movements.

Fourth, another descriptive measure of sensory sampling, the angle of the vector connecting the base and the tip of the sensory appendage in arena coordinates, was calculated (Figure 4A). The circular sector covered by the tip is summarized in Figure 4B (relative to the body axes). During backward locomotion, these circular sectors were clearly larger and further apart, away from the insect body axes, for the antennae, with the cerci showing a consistent, yet not statistically significant, trend.

**Figure 6:**
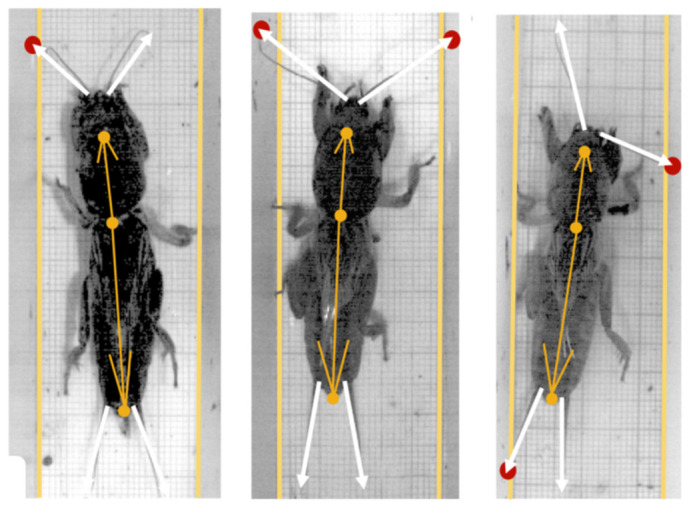
Representative snapshots from video sequences showing wall contact events by antennae and cerci (marked with red dots).

In a further attempt to quantify and compare the use of the sensory appendages for acquiring information about the topology of the environment during forward and backward locomotion, we calculated the fraction of time in a walking bout during which the tip of an antenna or cercus was in contact with the arena walls (Figure 5 and Figure 6). While the data presented in Figure 5D show much variation, mostly during backward locomotion, antennae wall contact clearly increased during backward locomotion compared with forward walking. Several insects also showed increased cerci contact during backward locomotion, yet on average, the difference was not consistent.

## 4. Discussion

Our findings suggest direction-dependent differences in the deployment of antennae and cerci during mole cricket locomotion. During backward walking, these insects exhibit specific behavioral adaptations that facilitate sensory sampling via their appendages, thereby maximizing relevant tactile input. Specifically, antennal trajectories extend further posteriorly relative to the body axis, while increased abdominal and possibly active cercal movements enhance the probability of posterior tactile sampling.

These observations support the idea, recently presented by Yuval et al. [28], that backward locomotion is not simply “forward control run in reverse”. Rather, it appears to involve a distinct sensorimotor configuration in which the insects adjust their sensory sampling to match the direction of travel. Such adjustments are likely to enhance the availability of tactile information relevant for locomotor control when animals move through confined spaces where environmental geometry must be inferred locally [1,2].

The observed repositioning of the antennae during backward locomotion suggests that these appendages continue to play an important role in sampling nearby boundaries even when the direction of progression is reversed. In our experiments, antennal trajectories extended posteriorly relative to the body axis, frequently reaching toward the tunnel walls as far back as possible. This configuration may allow the insect to maintain information about wall position while retreating, thereby helping stabilize body orientation and maintain alignment within the corridor. Such directional modulation of antennal deployment is consistent with the idea that insects actively adjust tactile sampling in order to extract behaviorally relevant spatial information [9].

The cerci occupy a particularly informative position during backward locomotion because they become the first appendages to encounter environmental boundaries. Given their relatively limited range of motion, the cerci may be used passively, encountering tactile stimuli as a result of body or abdominal movements. Our observations suggest that backward walking is associated with active cercal movements relative to the body axis together with occasional increases in side-to-side abdominal motion. Although statistical significance was not demonstrated in all comparisons of cercal movement, taken together, this behavior may suggest an increased potential for the cerci to encounter obstacles and possibly also come into contact with the tunnel walls. Such contacts could provide information about the proximity and orientation of nearby boundaries, thereby contributing to collision avoidance, corridor centering and the localization of bifurcation sites during retreat.

Although the cercal system has traditionally been studied in the context of escape responses to air currents [16,17,19], the behavioral patterns observed here suggest that these appendages may also participate in tactile sensing during locomotion. In narrow tunnels, where posterior contacts are likely to occur first during backward movement, cercal signals may provide early information about environmental constraints encountered along the direction of travel. Within this context, the cerci may function as leading-edge mechanosensors that complement the wall-referenced sampling provided by the antennae.

Backward locomotion is commonly recruited in insects when forward progression is impeded. Work on Drosophila has provided a mechanistic entry point to the control of walking direction, identifying descending command-like neurons whose activity is required and sufficient for backward walking under relevant sensory conditions [31]. Complementary findings show that mechanosensory cues such as touch can drive direction reversal through identified ascending pathways that recruit backward-walking circuits, supporting retreat from dead ends and obstacles in darkness [32]. Two recent and extensive reviews dedicated to insect walking and motor control [26,33] provide details of our current understanding of both behavioral aspects and underlying neuronal mechanisms of backward walking. Although the control mechanisms underlying backward locomotion in mole crickets remain unknown, the present results suggest that tactile signals originating from both antennae and cerci may contribute to maintaining or regulating retrograde motion.

It should be noted that the present study focused on appendage kinematics and wall-contact events in a simplified tunnel-like arena. While the observed differences between forward and backward walking are consistent with adaptive modulation of tactile sensing, additional experiments will be required to determine the causal contributions of each appendage. Manipulations that selectively restrict antennal or cercal movements and inputs could help clarify their respective roles in maintaining stability, avoiding collisions, and regulating locomotor direction. Furthermore, experiments conducted in more complex tunnel geometries may reveal additional sensorimotor strategies that are not expressed in straight corridors.

## 5. Conclusions

Taken together, the present findings are consistent with the hypothesis that backward locomotion in mole crickets involves contributions from both anterior and posterior mechanosensory appendages. Antennae may provide boundary-related tactile information associated with body orientation, whereas cerci may contribute information about environmental constraints encountered during retreat. These patterns are consistent with a possible role for these appendages in backward movement within confined subterranean environments.

## Data Availability

The original contributions presented in this study are included in the article/Supplementary Material. Further inquiries can be directed to the corresponding author.

## Supplementary Materials

The following supporting information can be downloaded at: https://www.mdpi.com/article/10.3390/insects17060564/s1.
